# Resistance of Subtype C HIV-1 Strains to Anti-V3 Loop Antibodies

**DOI:** 10.1155/2012/803535

**Published:** 2012-04-02

**Authors:** David Almond, Chavdar Krachmarov, James Swetnam, Susan Zolla-Pazner, Timothy Cardozo

**Affiliations:** ^1^Department of Pharmacology, New York University School of Medicine (NYSoM), New York, NY 10016, USA; ^2^Public Health Research Institute and New Jersey Medical School, University of Medicine and Dentistry of New Jersey, Newark, NJ 07101, USA; ^3^Department of Pathology, New York University School of Medicine (NYSoM), New York, NY 10016, USA

## Abstract

HIV-1's subtype C V3 loop consensus sequence exhibits increased resistance to anti-V3 antibody-mediated neutralization as compared to the subtype B consensus sequence. The dynamic 3D structure of the consensus C V3 loop crown, visualized by *ab initio* folding, suggested that the resistance derives from structural rigidity and non-**β**-strand secondary protein structure in the N-terminal strand of the **β**-hairpin of the V3 loop crown, which is where most known anti-V3 loop antibodies bind. The observation of either rigidity or non-**β**-strand structure in this region correlated with observed resistance to antibody-mediated neutralization in a series of chimeric pseudovirus (psV) mutants. The results suggest the presence of an epitope-independent, neutralization-relevant structural difference in the antibody-targeted region of the V3 loop crown between subtype C and subtype B, a difference that we hypothesize may contribute to the divergent pattern of global spread between these subtypes. As antibodies to a variable loop were recently identified as an inverse correlate of risk for HIV infection, the structure-function relationships discussed in this study may have relevance to HIV vaccine research.

## 1. Introduction

Subtype C infections now represent the majority of HIV-1 infections worldwide [1], suggesting greater *in vivo *or host-pathogen fitness. By contrast, in direct *in vitro *competition assays, R5 subtype B isolates outcompete R5 subtype C isolates [2], suggesting greater *in vitro* infective fitness. Thus, more rapid *in vivo *spread of subtype C infections may be occurring despite an apparent greater *in vitro *fitness of subtype B.

Differential susceptibility to human antibody-mediated neutralization could result in differing extents of global spread between different subtypes. The V3 loop is often referred to as the principal neutralizing determinant of HIV-1 viruses as several of the early and recent studies describing human antibodies that could neutralize HIV-1 were dominated by anti-V3 loop antibodies [3–6]. Indeed, several observations suggest a conformational or functional difference between subtype B and subtype C V3 loops [7], but the nature of the difference has not been elucidated. The V3 loop is also the site of CCR5 and CXCR4 engagement, a necessary determinant of virus entry [8–13]. Thus, antibody neutralization determinants and infective determinants coincide to the same location on the HIV-1 envelope glycoprotein surface, and disturbances to one are likely to affect the other.

**Figure 1:**
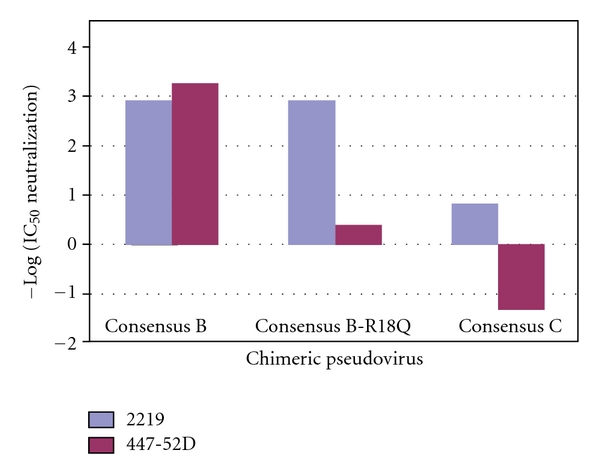
Neutralization of chimeric psVs by antibodies 447-52D (purple) and 2219 (blue). The psV consist of the SF162 strain with its V3 loop replaced by the indicated V3 loop sequence. Consensus B-R18Q is a psV consisting of the consensus subtype B V3 loop sequence with position 18 in the V3 loop mutated from a subtype B consensus arginine to a subtype C consensus glutamine. IC_50_ is the amount of antibody in ug/mL required to achieve 50% neutralization. The negative base 10 logarithm of the IC_50_ has been plotted for easier comparison: higher, positive bars towards the top of the graph indicate strong neutralization by the antibody. Antibodies were only tested to a concentration of 20 ug/mL for neutralization, so the negative value of 1.3 on the plot is maximal and indicated no detectable neutralization by the antibody. Adapted from [14].

A comparison of antibody-mediated neutralizations of SF162 chimeric psVs carrying the consensus subtype C V3 loop sequence (conC) to those for the consensus subtype B V3 loop sequence (conB) by two different broadly neutralizing anti-V3 loop monoclonal antibodies (mAbs) previously demonstrated that ConC has substantially more resistance to neutralization mediated by both antibodies (Figure 1) [14]. Each of those mAbs (i.e., 2219 and 447-52D) has been crystallographically confirmed to have distinct V3 loop binding modes [15, 16]. Poorly characterized and variable V3 loop surface exposure features were controlled in these experiments using a previously established approach in which psVs are constructed to express different sequences of the V3 loop within the same SF162 Env background [17]. SF162 is sensitive to antibody-mediated neutralization and in this setting provides a relatively constant level of V3 loop exposure and a minimal Env variability background across the different chimeric psVs. Differences in neutralization between two mutants differing only in their V3 loop sequences should therefore be caused by structural differences in the V3 loop itself. Thus, some portion of the subtype C virus resistance to anti-V3 antibody-mediated neutralization maps to the V3 loop itself and not to structural effects outside the V3 loop or to surface exposure differences between subtype B and subtype C.

Previous analysis of the 3D interaction surface of 2219 and 447-52D bound to the V3 loop divides the V3 loop into amino acid positions that comprise the Ab “epitope” and those that do not [14]. The 3D structure of the epitope or Ab-bound surface can be divided into two functionally distinct categories. The first category is amino acids that were found to comprise the neutralization epitope: these are defined as tight (usually electrostatic), substitution-intolerant complementarities between pockets on the antibody surface, and specific V3 amino acid side chains that restrict the neutralization activity of the antibody. These amino acids were identified as R18 for 447-52D and K10-I12-Y21 for antibody 2219 [14], where the number following each amino acid single-letter designation refers to the position of that amino acid in the V3 loop starting with the disulfide-bonded cysteine as number 1. Mutation of any of these amino acid side chains disrupt Ab-mediated neutralization strongly.

The second category is the rest of the epitope or Ab-bound 3D molecular surface: mutation-tolerant contacts between loosely bound V3 side chains or the V3 loop backbone and the antibody binding surface that depend on the overall tertiary shape of the bound V3 loop conformation. We define these areas as mutation insensitive, “epitope-independent” locations for the purposes of this study, although technically all atoms contacting the Ab comprise the epitope and these locations can indirectly influence the epitope.

The conC sequence differs from the conB sequence in the former, substitution-intolerant type of contact. Thus, with a Q18 present in ConC versus R18 in ConB, the chimeric psV carrying the conC loop was shown to be predictably resistant to antibody 447-52D (up to 20 ug/mL tested, Figure 1). However, when this important contact was abolished in the conB sequence (by an R-to-Q mutation at position 18 (R18Q), antibody-mediated neutralization was reduced but not abolished, indicating that the observed resistance to antibody-mediated neutralization does not depend completely on this specific neutralization epitope contact (Figure 1). Conversely, the conC sequence *does not *differ from the conB sequence in neutralization epitope contacts for antibody 2219 (K10, I12, and Y21), but conB is sensitive to 2219 antibody-mediated neutralization while conC is partially resistant. The magnitude of this epitope-independent resistance for 2219 is approximately of the same magnitude as that inferred for 447-52D. Thus, for both antibodies, a substantial epitope-independent structural mechanism, present in conC but not in conB, appears to underlie resistance to anti-V3 antibody-mediated neutralization of the subtype C virus.

We therefore hypothesized that we could visualize the 3D structural basis of the epitope-independent differential resistance observed between the subtype B and subtype C V3 loops by correlating previously published psV neutralization measurements with dynamic 3D structural visualization of several carefully constructed V3 loop mutants. In order to do this, we needed a minimum of 2 Abs with crystallographically confirmed differences in their targeted V3 crown epitopes, so Abs 447-52D and 2219 were chosen, but we expect these results to apply generally to all V3 crown-targeted Abs.

## 2. Results

### 2.1. The Segment at Positions 12 to 14 of the Consensus C V3 Loop Crown Has a Rigid, Non-*β*-Strand Conformation

**Figure 2:**
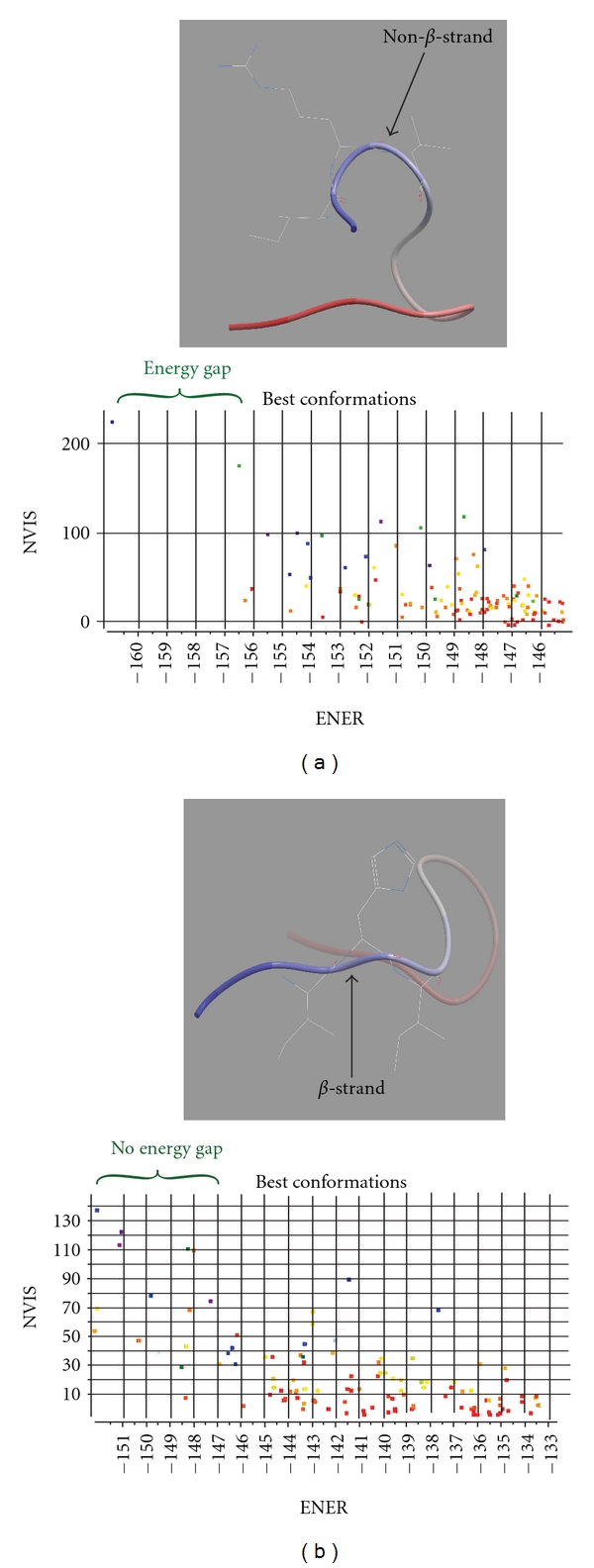
(a) *Top: *lowest energy conformation of the subtype C V3 loop crown from V3 loop amino acid positions 10 to 22. The structure is shown in ribbon representation colored in a gradient from the N-terminal residue at position 10 (dark blue) to the C-terminal residue at position 22 (colored dark red). The side chain conformations of Ile12, Arg13, and Ile14 are shown in full-atom wire representation. Their backbone curvature and fanned orientation are inconsistent with *β*-strand secondary structure. *Bottom: *plot of 180 lowest energy conformations from the folding simulation. *x*-axis (ENER): energy score ranging from lowest at the left to higher at the right. *y*-axis (NVIS): number of visits by the simulation to the indicated conformation. For example, the lowest energy conformation was found again and again by the search over 150 times. The presence of a 4 kcal simulation energy unit gap in the subtype C simulation indicates a rigid structure as a 4 kcal energy barrier prevents exit from the lowest energy conformation most of the time on the biological time scale. (b) *Top: *lowest energy conformation of the subtype B V3 loop crown from positions 10–22 depicted as in A. The extended linear conformation of Ile12, Arg13, and Ile14 with alternating directions for the side chains is typical of canonical *β*-strand structure. *Bottom: *plot of 180 lowest energy conformations from the folding simulation as in A. Many conformations are present near the lowest energy conformation in this subtype B simulation predicting a flexible structure that flickers between ~10 conformations all the time (is flexible) on the biological timescale.

Several recent studies have visualized snapshots of the 3D structure of the V3 loop by NMR and crystallography [15, 16, 25]. One common feature of mAbs 2219 and 447-52D is that both bind positions 12 to 14 of the V3 loop in an antiparallel *β*-sheet fashion. This result may be applicable to most broadly neutralizing anti-V3-loop antibodies, as their linear epitopes overlap, for the most part, at the N-terminal *β*-strand (positions 12 to 14) of the V3 loop (M. Gorny, personal communication). The *β*-strand at positions 12–14 of the V3 loop appears to be a common structural feature required for recognition and function of anti-V3 antibodies. Since this region makes mostly backbone (non-side-chain) contacts with the V3 antibodies, the protein backbone conformation of this region (i.e., *α*-helical, *β*-strand, etc.) is likely to influence anti-V3 antibody-mediated neutralization. We therefore examined this region carefully for backbone conformational differences between neutralization-resistant conC and neutralization-sensitive conB. *ab initio* peptide folding algorithms have previously been shown to be capable of predicting the flexibility and conformational preferences of the V3 loop crown, recapitulating crystallographic forms (See Supplementary Figure 1 in the Supplementary Material available online at doi:10.1155/2012/803535) and demonstrating that the V3 loop crown from positions 10 to 22 of the V3 loop behaves as an autonomously folded, but flexible, domain [18–20]. Folding a peptide identical in sequence to the conC V3 crown from positions 10 to 22 shows that the peptide backbone prefers a rigid, non-*β*-strand structure at positions 12 to 14 (Figure 2). In contrast, a peptide identical in sequence to amino acids 10–22 of the conB V3 crown backbone adopts a flexible conformation with clear *β*-strand character at positions 12 to 14 and overall a clear *β*-hairpin fold (Figure 2). The key positions in the conB sequence consistently adopt *ϕ*-*φ* angles typical of a Type II beta-hairpin at the V3 GPG sequence, while these are lost in conC. The rigidity and non-*β*-strand structure of the 12–14 V3 segment in the subtype C V3 crown may present an energetic barrier to peptide deformations required for antibody-mediated neutralization, specifically bending of the structure into and out of a neutralization relevant *β*-strand conformation. Thus, these results suggest the following epitope-independent structure-activity relationship: anti-V3-loop-antibody-mediated neutralization depends on a flexible *β*-strand at positions 12 to 14 in the V3 loop.

### 2.2. Substitutions in the V3 Antibody Binding Site Affect the Rigidity and *β*-Strand Conformation of This Area

We began by focusing on the structural effects of mutations at the key positions 13 and 14 of the subtype C V3 loop by folding a series of V3 loop crowns with point mutations at these positions (Table 1). Position 12 was avoided because it is part of the neutralization epitope for 2219 and such mutants would therefore obviate a standardized assessment of the non-epitope-dependent effects across mutants. *ab initio* folding of conC mutated in the 14th position of the V3 loop from Ile to Met (I14M) mildly increased the flexibility of the V3 crown but retained a strong *β*-hairpin conformation. *ab initio* folding of an I14V conC mutant restored full flexibility and 2/3 *β*-strand character to this local region. Folding of I14L demonstrated a non-*β* strand, partly *α*-helical conformation. Interestingly, we previously demonstrated that *α*-helical conformations in this region are sufficiently disruptive to abolish infectivity of the virus [20], suggesting that I14L has the strongest resistance to antibodies in this dataset but may also be the least infective construct. I14F re-established two-thirds of the *β*-strand in the 12 to 14 region, but did not preserve a *β*-hairpin as the C-terminal strand of the V3 crown did not contact the N-terminal strand and the overall structure remained somewhat rigid.

Comparison of this folding data with neutralization data from *in vitro* chimeric psVs with the same mutations showed that loss of 2219 antibody-mediated neutralization correlated with the loss of both *β*-strand character and structural flexibility, while reduction of 2219 neutralization is associated with the loss of either factor independently (Table 1). As noted previously, all tested chimeric psVs from Table 1 preserve the key 2219 binding epitope side chains as a control feature, so increased resistance to antibody-mediated neutralization appears to correlate with loss of *β*-strand or *β*-hairpin conformation in the V3 loop and/or loss of structural flexibility at positions 12 to 14 in an epitope-independent manner.

Mutations anywhere in the V3 crown can affect the folding of the whole crown, and as such simulations were performed for mutations outside of positions 13 and 14. *In silico *mutations at positions 18 and 22 in conB did not alter the folding significantly (Table 1). A conC T19A *in silico *mutation did alter overall folding somewhat, resulting in partial restoration of flexibility and full restoration of *β*-strand character at 12–14. These results also correlated with the previously noted resistance of *in vitro* chimeric psVs to 2219 antibody-mediated neutralization, so changes outside the key Ab-targeted region can indirectly affect folding, and the observed effect is tertiary and not specifically dependent on any amino acid position.

### 2.3. The Epitope-Independent Effect May Be General to a Wide Variety of Anti-V3 Antibodies

When tested with 14 broadly neutralizing anti-V3 antibodies derived from donors infected with subtypes A and B, the conC chimeric psV was neutralization resistant to all of the mAbs to a much greater degree than the conB chimeric psV (Table 2). A non-V3 Ab—b12—did not show the same magnitude of effect. In the panel, 447-52D and 2219 are known to have distinct epitopes, and it is likely that many of the other mAbs have distinct epitopes as well. The common resistance of conC to all these different antibodies suggests an epitope-independent structural resistance to neutralization residing in the V3 loop.

## 3. Discussion

The experimental results described here indicate that a significant fraction of the anti-V3 antibody-mediated neutralization resistance of the conC sequence maps directly to the antibody-binding domain of the V3 crown. Furthermore, the epitope-independent structural feature by which the subtype C V3 crown resists neutralization by a variety of anti-V3 antibodies appears to be a rigid N-terminal non-*β*-strand conformation at positions 12 to 14 of the V3 loop. This effect is exclusive of the more commonly observed mechanism of antibody escape, that is, mutations of key neutralization epitope side chains, such as R18Q for 447-52D which we have shown results in a distinguishable, antibody-specific resistance. The combination of the loss of key neutralization epitope amino acid side chains with rigidity or non-*β*-strand structure results in total resistance of the psV bearing these V3 loop properties to neutralization by the antibody in question. Since these are intrinsic features of the V3 loop sequences, this phenomenon would apply to circulating viruses bearing these properties in their V3 loops as well, specifically subtype C viruses. Our dissection of the effects of single V3 loop point mutations shows that the effects of each point mutation is complex, and the backbone effect is combinatorial to all of the V3 loop positions simultaneously. Thus, no single amino acid position is solely responsible for the conC structural phenomenon. The convergence of three completely independent sets of data—(1) known crystallographic structures of V3 peptides bound to antibody, (2) patterns of psV neutralization, and (3) validated *ab initio* folding simulations—strongly supports these conclusions.

Our observation suggests that a flexible, *β*-strand structure at positions 12 to 14 is required for anti-V3 antibody-mediated neutralization, and indeed this region is bound by many anti-V3 antibodies. Nevertheless, it cannot be concluded that antibody binding alone underlies this structure-activity relationship. Neutralization is a multistep process with antibody binding being only one step. One can imagine a rigid V3 loop crown shape that is perfectly complementary to an antibody-combining site and therefore binds the antibody* in vitro,* but the virus may nevertheless be neutralization resistant due to the effects of this selfsame rigidity at other steps in the neutralization process. For example, neutralization-relevant V3 loop interactions with several other surfaces of gp120 may be affected by the rigidity in the V3 loop crown. For this reason, it is possible that structural rigidity in the V3 loop crown may also influence neutralization by non-V3-targeted antibodies by inhibiting intermediate conformations involving the V3 loop in the series of conformational changes that likely comprise the overall neutralization process. Indeed, the conC psV exhibits mildly increased resistance to the non-V3 Ab b12 (Table 2).

The unique resistance of conC to a wide variety of subtype A and subtype B derived anti-V3 antibodies may be informed by the observation of low variability of the consensus C sequence in circulating subtype C strains [26]. If neutralization via the V3 loop is a strong selection pressure on circulating subtype C viruses, then infective V3 loop sequences harboring resistance to anti-V3-loop-antibody mediated neutralization might be observed at a higher rate and exhibit fewer escape mutations (vary less in sequence). As a corollary, subtype A and B derived anti-V3 antibodies may not be very effective as vaccine tools to combat subtype C, an observation that has previously been suggested for subtype B [27]. Different strategies for interrogation of subtype C infected HIV+ sera may be required to uncover novel, effective neutralizing antibody responses to this subtype. On the other hand, a diversity of V3 loop sequences are present in subtype C in addition to the dominant conC and conC-like sequences. It is unlikely that the effect we have observed is universal in subtype C, and some subtype C strains may exhibit V3 loop flexibility or encode compensatory changes in other parts of gp120 to afford efficient neutralization by anti-V3 antibodies.

The psV neutralization data correlates strongly with the results of the folding simulations. This correlation extends previous observations suggesting that the 12 *β*-hairpin residues (positions 10 to 22) of the V3 crown—including the currently known anti-V3 antibody combining sites—are sufficiently flexible in situ in the V3 loop to behave essentially as free peptides or as an autonomously folded subdomain [18–20]. *Ab initio* folding simulations of the V3 loop crown may therefore visualize at low resolution the dynamic structural ensemble of some V3 loop crowns *in silico*, a potentially important high-throughput capability for mapping structure-(neutralization) activity relationships in the V3 loop crown. It should be noted that the identification of two properties that are more easily assessed in a dynamic ensemble of many conformations—secondary structure and rigidity—facilitated the comparative interpretation of our library of folding data. Observing the structural tendency at a very specific location—positions 12 to 14 in the V3 loop—also facilitated the study. More subtle and global structural patterns, including overall fold assignment and absolute energetic stability for the whole domain, may be more difficult to discern and assess across different foldings for the purpose of correlations with experiments.

The strong correlation of the psV neutralization measurements with observed structural features in the folding simulations also establishes the SF162 chimeric psV system as one that provides, even to fine resolution, a consistent virologic background across multiple experiments and V3 loop sequence variations. The combination of chimeric psV neutralization measurements with *ab initio* folding simulations allows detailed quantitative *dynamic* structure-neutralization activity relationships to be mapped out for the V3 loop. Such studies would be difficult with crystallography, which is low throughput and does not evaluate dynamic structure.

Antibody epitopes in the V3 loop may occur broadly in HIV-1 viruses, but antibodies appear to be limited in accessing these epitopes presumably due to the effects of “masking” glycans and nearby variable domains [17]. In this work, the comparison of neutralization tests with folding simulations may have revealed an obscure structural explanation for epitope-independent variations in antibody-mediated neutralization. The fact that some of the resistance to antibody-mediated neutralization maps directly to the antibody binding area of the V3 loop influences the view of masking: some of the observed masking of V3 loop epitopes may be intrinsic to the V3 loop itself and not due to outside factors such as glycans and the other variable loops of gp120, although both factors are likely operative to different degrees in any given strain. The approach described here could potentially be modified to “localize” the masking of the V3 loop epitope in primary HIV-1 isolates, at least to V3 loop or “outside-V3 loop” locations, by identifying anti-V3 loop antibody-resistant primary isolate sequences in which this “local masking” is present.

HIV-1 strains that evolve to partially mask receptor interacting surfaces in order to hide those vulnerable surfaces from the immune system trade a loss of infective efficiency for a gain in camouflage protection. The coincidence of determinants for infection (chemokine receptor binding surfaces) and immune detection (broadly neutralizing epitopes) in the V3 loop likely requires such a tradeoff for best viral fitness. Since a wide variety of anti-V3 antibodies appear to adhere to the antibody resistance mechanism described here, the work suggests that this tradeoff is accomplished in subtype C partly by the adoption of V3 loop structural shapes that are inefficient for both antibody-mediated neutralization and coreceptor binding, that is, rigid non-*β*-strand conformations. As the same viral mechanisms that produce this feature of the V3 loop beget the structural features of the other four variable loops, it is likely that the same tradeoff is exploited by viral evolution for functional regions of the V1/V2, V4, and V5-loops. If anti-variable loop antibodies play a significant enough role in the human protective response to circulating HIV-1 strains, the phenomenon we have described here may explain the concurrent diverging observations of decreased fitness (poorer receptor usage) and increased natural spread (successful immune evasion) in subtype C. Indeed, the recent identification of anti-V2 loop Abs as the only known inverse correlate of risk for HIV infection suggests that antibodies to variable loops indeed play a significant role in the human protective response from circulating HIV-1 strains [28].

## 4. Methods

### 4.1. Dynamic Structural Characterization of the V3 Crown

In order to efficiently correlate 3D structure with neutralization patterns, the visualization of the dynamic conformational landscape of various mutants of the V3 loop crown *in silico* is needed. Interestingly, the identical sequence—V3^MN^—has been solved in complex with the two different antibodies and adopts different *β*-strand structures in the two different environments despite the identical sequence. These essentially represent biologically relevant snapshots of two conformations out of many in V3^MN^'s dynamic conformational ensemble. We previously reported that a state-of-the-art *Ab initio* peptide folding algorithm could accurately reveal the conformational landscape and dynamic tertiary structure of the V3 loop crown by analyzing whether the two different forms of V3^MN^ seen in the crystallographic structures were recapitulated [18]. *ab initio *folding of residues 10 to 22 of the V3 crown recapitulated the two forms and demonstrated the relationship between them (the 2219 form is the lower energy more prevalent form). Since the input to the folding is only the amino acid sequence, this algorithm can thus at least faintly visualize *in silico *the biologically relevant dynamic ensemble of any V3 loop crown sequence from position 10 to 22. This capability was later verified by an independent group [19]. This result also suggests that this portion of the V3 crown behaves similarly to an autonomously folded, free, unconstrained peptide, since the folding simulation used no constraints on the two stems of the peptide and the folding qualitatively recapitulated a form seen experimentally in the V3 loop in situ in gp120 [6]. Finally, this technology was used to engineer an *α*-helical V3 loop crown and demonstrate that it loses infectivity [20]. *Ab initio *folding of residues 10 to 22 of the V3 crown of all the psVs used in this study was thus used to evaluate the flexibility and conformational preferences of V3 loop crown structures *in silico*.

All folding simulations and analysis were performed with ICM software (MolSoft LLC, La Jolla, CA, USA) as previously described [21]. This algorithm has previously been shown to predict experimentally verified peptide structures up to 23 residues in length within the error accuracy limit [22]. As a starting point for each folding run, fully extended conformations of full-atom (including hydrogens) models of each indicated V3 loop crown sequence (positions 10–22) were generated. The total number of energy evaluations during the MC run was based on the number of free variables (number of free standard torsion angles). Each MC run took 2-3 hours of CPU time on a 3.00 Ghz Dual-Core Intel Xeon Processor. The exact script for the foldings and all the folding data are available upon request from the corresponding author. 

All the data on chimeric psV construction and neutralization assays were previously reported in the literature [14, 17], except those repeated for confirmation in this study or those in Table 2. In all cases, the same method was used. Briefly, each chimeric psV was constructed to contain a different V3 loop sequence grafted in to replace the V3 loop in the SF162 Env, where the V3 loop is relatively accessible (“unmasked”) [17]. Thus, the observed differences in psV neutralization elicited by each mAb maps to differences in the V3 loop sequences. The V3 chimeric SF162 psVs were constructed with V3 mutations introduced as indicated in the text in the consensus subtype B or consensus subtype C V3 loop, or V3 chimeric SF162 psVs were constructed in which the SF162 V3 loop was replaced with the V3 loop consensus sequences from subtypes A1, B, C. The neutralization by mAbs 447-52D and 2219, as well as other Abs, of each of these psVs was assessed using methods previously described [23]. Briefly, neutralizing activity was determined with a single-cycle infectivity assay using psVs generated with the *env*-defective luciferase-expressing pNL4-3.Luc.R-E- plasmid v [24] pseudotyped with the SF162 V3 variants described above. The psVs were incubated with serial dilutions of mAbs for 1.5 hour at 37°C and then added to CD4+CCR5+U87 target cells plated in 96-well plates in the presence of polybrene (10 mg/mL). After 24 hrs, cells were refed with RPMI medium containing 10% FBS and 10 *μ*g/mL polybrene, followed by an additional 24–48 hr incubation. Luciferase activity was determined 48–72 hrs postinfection with a microtiter plate luminometer (HARTA, Inc.) using assay reagents from Promega, Inc. Geometric mean titers for 50% neutralization (GMT_50_) were determined by interpolation from neutralization curves and are averages of at least three independent assays.

## Figures and Tables

**Table 1:** 2219 antibody-mediated neutralization of psVs constructed from SF162 with the V3 loop replaced by the consensus C or consensus B V3 loop sequence with and without the indicated point mutations in the “Sequence” column. *In vitro* measured strong, weak, or no neutralization is indicated along with the IC_50_ (ug/mL) in the “Neutralization” column on the right. Numbering of mutated residues is from the beginning of the V3 loop with the starting cysteine being residue number 1 so that D25E (V3 loop numbering) is the same as D322E (numbering of residues from N-terminus of gp120). The “Flex” column is the structural flexibility of the V3 crown from positions 10 to 22 as assessed by *ab initio *folding: +++ indicates no energy gap and many conformations near the energy minimum suggesting a flexible structure; ++, +, and −indicate a spectrum of energy gaps of <2 U slightly more than the standard error of the energy function suggesting a partly flexible structure; −− indicates an energy gap >2 U indicating a rigid conformation. The “*β*-hairpin” column is the *β*-strand character of positions 12 to 14 as assessed in the same *ab initio *folding: +++ indicates that all three residues from 12 to 14 adopt canonical *β*-strand *ϕ* and Ψ angles in the lowest energy structure; ++ indicates that two of the three residues from 12 to 14 adopt canonical *β*-strand *ϕ* and Ψ angles; + indicates that two or more of the residues from 12 to 14 adopt canonical *β*-strand *ϕ* and Ψ angles, but that the overall structure does not form a *β*-hairpin. – and −− indicate that residues from 12 to 14 adopt canonical non-*β*-strand *ϕ* and Ψ angles.

Sequence	Flexibility	*β*-Hairpin	Naturalization
			(IC_50_ *μ*g/mL)
Consensus B	+++	+++	Strong (0.001)
B-R18Q	+++	+++	Strong (0.001)
B-T22A	+++	++	Strong (0.002)
C-I14V	++	+++	Strong (0.01)
C-T19A	+	+++	Weak (0.02)
C-I14M	++	−−	Weak (0.03)
C-I14F	−	++	Weak (0.1)
Consensus C	−−	−−	None (0.15)
C-D25E	−−	−−	None (0.15)
C-I14L	−	−−	None (0.15)

**Table 2:** IC_50_s (ug/mL) of 15 different antibodies (columns) derived from subtype B and subtype A infected patients neutralizing the infectivity of psVs containing the subtype C and B V3 sequences in the SF162 Env backbone. For comparison, the IC_50_ values for a non-V3 Ab (b12) are as follows: clade B cons. (JR-FL) = 0.009 ug/mL; clade C cons. = 0.02 ug/mL, all others untested; which does not show as dramatic a difference in neutralization between the two psVs. 135 MPL23a is a subtype C primary isolate and is included as an example of IC_50_ values in non-neutralization-sensitive (“masked”) backgrounds. IC_50_ values are font-type coded as follows: bold >0.1 ug/mL; italic <0.1 ug/mL; bold/italic <0.01 ug/mL.

V3 sequence	Anti-V3 mAbs from clade B infected patients								Anti-V3 mAbs from clade A infected patients						
	**2412**	**4117**	**2442**	**4148**	**2456**	**447**	**2191**	**2219**	**2128**	**3074**	**2557**	**2558**	**3019**	**3224**	**2601**
c1. B cons (JR-FL)	***0.0009***	***0.0055***	***0.0018***	***0.0053***	***0.0039***	***0.00054***	***0.0023***	***0.0012***	***0.0013***	***0.0055***	***0.0037***	***0.0049***	***0.0027***	***0.0051***	0.12
clade A1 cons	2.81	14.4	*0.027*	*0.068*	*0.083*	0.58	*0.043*	*0.029*	**>20**	*0.015*	*0.025*	*0.038*	*0.032*	*0.044*	*0.029*
clade C cons	**>20**	**>20**	**>20**	2.5	0.44	**>20**	0.59	0.15	**>20**	0.2	0.16	0.2	0.25	1.8	0.17
IC_50 _ratios B/C	>22,000	>3,600	>11,000	470	113	>37,000	257	125	>15,000	36	43	41	93	345	1.4
135 MPI 23a*	**>20**	**>20**	**>20**	**>20**	**>20**	**>20**	**>20**	**>20**	**>20**	8.0	**>20**	77.0	93.0	**>20**	**>20**

*Primary subtype C isolate.

## Abbreviations

HIV: Human immunodeficiency virus.
